# Cytomegalovirus Reactivation in Adult Recipients of Autologous Stem Cell Transplantation: a Single Center Experience

**DOI:** 10.4084/MJHID.2015.049

**Published:** 2015-08-24

**Authors:** Omar Al-Rawi, Fawzi Abdel-Rahman, Rula Al-Najjar, Husam Abu-Jazar, Mourad Salam, Mustafa Saad

**Affiliations:** 1 Stem cell & bone marrow transplantation program, King Hussein Cancer Center, Amman, Jordan; 2 Division of Infectious Diseases, Prince Sultan Cardiac Center, Prince Sultan Military Medical City, Riyadh, Saudi Arabia

## Abstract

**Introduction:**

Cytomegalovirus (CMV) reactivation and infection are well-recognized complications after allogeneic stem cell transplantation (SCT). Only a few studies have addressed CMV reactivation after autologous SCT (ASCT).

**Methods:**

We retrospectively reviewed medical records of 210 adult patients who underwent ASCT for lymphoma or multiple myeloma (MM) at a single center from January 1^st^, 2007 until December 31^st^, 2012. All patients were monitored weekly with CMV antigenemia test till day 42 after transplantation, and for 2 months after last positive test in those who had any positive CMV antigenemia test before day 42.

**Results:**

Thirty-seven (17.6%) patients had CMV reactivation; 23 patients had lymphoma while 14 had MM as the underlying disease. There was no difference in the rate of CMV reactivation between lymphoma and MM patients (20% versus 14.7%, *P* = 0.32). The majority of the patients were treated with ganciclovir/valganciclovir, all patients had their reactivation resolved with therapy, and none developed symptomatic CMV infection. None of the patients who died within 100 days of transplantation had CMV reactivation. Log-rank test showed that CMV reactivation had no effect on the overall survival of patients (P values, 0.29).

**Conclusion:**

In our cohort, CMV reactivation rate after ASCT was 17.6%. There was no difference in reactivation rates between lymphoma and MM patients. With the use of preemptive therapy, symptomatic CMV infection was not documented in any patient in our cohort. CMV reactivation had no impact on patients’ survival post ASCT.

## Introduction

Cytomegalovirus (CMV) reactivation and infection are known complications of allogeneic stem cell transplantation (SCT). Its incidence is more frequent after allogeneic SCT than after autologous SCT.^1^

Previous studies on CMV reactivation after autologous SCT (ASCT) showed an incidence of 30–40% in patients who were monitored by weekly CMV polymerase chain reaction (PCR) or antigenemia tests, and 1–13% in those monitored by clinical signs of CMV infection.^2^–^10^

The incidence of CMV reactivation following ASCT has not been carefully evaluated in subsets of patients with different underlying hematological malignancies such as multiple myeloma (MM) and lymphoma. Studies in this regard are sparse.

The prevalence of CMV immunoglobulin G (IgG) positivity in the population of the Eastern Mediterranean region is reported to be higher than that of the population of the western countries; Bazarbachi et al. reported a prevalence of 90% in the Eastern Mediterranean region compared to 60% in Europe.11

We performed this single-center study to evaluate the incidence of CMV reactivation in recipients of ASCT, to compare the incidence of CMV reactivation in patients with lymphoma versus multiple myeloma (MM), and to assess the outcome of these reactivations and their impact on transplant recipients’ survival.

## Patients and Methods

### Patients

We included all patients who had ASCT at King Hussein Cancer Center, Amman, Jordan, in the period between January 1^st^, 2007 and December 31^st^, 2012. Data were retrospectively collected from the patients’ medical records and the bone marrow transplantation program’s database. We collected information on patients’ demographics, underlying diseases, CMV status, conditioning regimens, CMV reactivation, presence of signs and symptoms of CMV infection, treatment modalities, CMV reactivation outcomes, and patients’ survival. Composed data entered into a computerized database and then were analyzed. The protocol was approved by the local Institutional Review Board; written informed consent was waived.

Locally developed guidelines were followed for the diagnosis and management of patients with CMV reactivation.

### Definitions

CMV reactivation was defined as a positive CMV antigenemia test in ≥5 cells/250,000 leukocytes examined, or if the test was positive in less than 5 cells on two or more consecutive occasions with unexplained cytopenias and/or liver enzyme elevation.

Cytopenias were defined as an absolute neutrophil count (ANC ) <1000 ×10^9^/L, platelet count <70 ×10^9^/L, and/or hemoglobin level less than 10 gm/dl. Patients were considered to have liver enzyme elevation if they developed values more than 1.5 times the upper limit of normal. These patients underwent careful evaluation for other potential causes of cytopenias and/or liver enzyme elevations such as drugs, and other viral infections.

CMV pneumonia was defined as the presence of interstitial infiltrates on chest radiographs accompanied by a histopathological demonstration of CMV in lung biopsy material. CMV gastrointestinal infection was defined as the presence of gastrointestinal symptoms accompanied by a histopathological diagnosis of CMV infection.

### CMV monitoring

All patients were routinely monitored for CMV reactivation on weekly basis after engraftment until day 42 post stem cell infusion, and those with positive CMV antigenemia test before day 42 were further monitored for 2 months after last positive test. Monitoring was done by testing for the presence of CMV pp65 antigenemia. CMV antigenemia assay is based on the detection of the CMV lower matrix protein pp65 in polymorphonuclear leukocytes by immunostaining with monoclonal antibodies.

CMV antigenemia testing was performed in duplicates^12^ using cytocentrifugation slides prepared of 2.5 × 10^5^ peripheral blood leukocytes per slide. Slides then were fixed with formaldehyde,^12^,^13^ and then stained with the immunofluorescence staining using monoclonal antibodies ppUL83 (pp65) blend (Argene, Biomerieux, Marcy l’Etoile, France).

### Antiviral prophylaxis and therapy

All patients were given prophylaxis with acyclovir 250mg/m^2^ intravenously every 8 hours from day minus 3 until white blood cell engraftment (absolute neutrophil count [ANC] > 500 ×10^9^/L for 2 consecutive days) when they were changed to oral acyclovir 400 mg every 12 hours. Routine CMV monitoring was started immediately after engraftment by weekly CMV antigenemia testing till day 42 post-transplant. Patients who developed a positive CMV antigenemia test but did not meet the criteria for CMV reactivation (i.e. more than 5 positive cells, or more than two readings less than 5 cells with unexplained cytopenias and/or liver enzyme elevation) had their prophylaxis changed to valacyclovir 1 gm orally every 8 hours.

Patients with CMV reactivation having adequate blood counts (ANC>1000 ×10^9^/L, and platelets >70 ×10^9^/L) were treated with ganciclovir 5mg/kg every 12 hours during the induction phase of therapy followed by 5mg/kg every 24 hours during the maintenance phase. Alternatevely, valganciclovir 900 mg was give orally twice a day during the induction phase followed by 900 mg and daily during the maintenance phase of therapy. On the other hand, patients who had cytopenias were treated with foscarnet at a dose of 90 mg/kg every 12 hours for induction followed by 90mg/kg every 24 hours for maintenance therapy.

The induction therapy was given initially for 7 days, and if repeat CMV antigenemia test became negative, patients were then switched to maintenance therapy for 10 more days. On the other hand, if positive results appeared on repeated testing, induction therapy was continued, repeat CMV antigenemia testing was done twice weekly, and patients were switched to maintenance therapy once the test turned negative.

### Statistical analysis

Descriptive statistics was performed on demographic data and clinical information of patients, showing counts and percentages for categorical data, and medians and ranges for continuous data. Chi-square test was used to compare categorical data as appropriate, depending on the assumptions required for each test. Overall survival was presented using Kaplan-Meier curves. Log-rank test was used to compare survival times. A P value of < 0.05 was considered statistically significant. All analyzes were performed using SAS version 9.1 (SAS Institute Inc, Cary, NC).

## Results

A total of 210 consecutive adult patients with lymphoma (55%) and MM (45%) who underwent ASCT were included. The majority of patients were females (58.6%), less than 50 years old (61.9%), had partial remission of their underlying hematological malignancy before transplantation (70%), and received acyclovir for prophylaxis (94.3%). Of the 210 patients, 205 (97.6%) were CMV IgG positive before transplantation while 10 (4.8%) were CMV IgM positive. All of the latter had a negative baseline CMV antigenemia test.

All patients received appropriate chemotherapy to treat the underlying hematological malignancy before being considered for transplantation. Patients with MM were treated with thalidomide and dexamethasone as first-line therapy; as for high-risk patients, cyclophosphamide was added to this regimen. Ten patients received bortezomib as a second-line therapy due to poor response to first-line chemotherapy. Patients with Hodgkin lymphoma were treated with ABVD (adriamycin, bleomycin, vinblastine, and dacarbazine), while those with diffuse large B-cell lymphoma were given R-CHOP (rituximab, cyclophosphamide, adriamycin, vincristine, and prednisone) as first-line therapy. Second-line chemotherapy in lymphoma patients was DHAP (dexamethasone, ara-C, and cisplatin), third-line was ICE (ifosfamide, carboplatin, and etoposide), and the fourth-line was mostly navelbine, or gemcitabine.

Three conditioning regimens were used according to the underlying disease. In the lymphoma group, 111 (96.5%) patients received BEAM (BCNU, etoposide, cytarabine, melphalan) chemotherapy regimen for conditioning, and 4 (3.5%) patients received TEAM (thiotepa, etoposide, cytarabine, melphalan). All patients with MM received melphalan 200mg/m^2^ as the conditioning regimen. All patients had white blood cell engraftment by day 15 of transplantation (median, 10 days). Overall mortality in our cohort was 23.8%, while day 100 mortality was 2.9%. Table 1 outlines clinical characteristics of the study cohort.

Overall, 37 (17.6%) patients had CMV reactivation. The median time to CMV reactivation was 31 days (range, 21–54 days). Some patients were continued on monitoring beyond day 42, because they had CMV antigenemia positivity during the first 42 days, so they were monitored for another two months from the last positive test.

At the time of reactivation, 35 (94.6%) patients were treated with ganciclovir or valganciclovir. Foscarnet was used to treat CMV reactivation in 2 (5.4%) patients due to baseline cytopenias (ANC<1000 ×10^9^/L, and/or platelets <70 ×10^9^/L). All patients had their CMV reactivation resolved with therapy, and none developed symptomatic CMV infection. The anti-CMV induction and maintenance therapies were given for a median of 8 and 10 days, respectively. The majority of side effects from therapy were cytopenias in the ganciclovir/valganciclovir treated patients, and electrolyte disturbances and renal impairment in the foscarnet treated patients. The overall mortality was 29.7% among patients with CMV reactivation. However, none of the patients who died within 100 days of transplantation had CMV reactivation. Table 2 summarizes the characteristics of patients who developed CMV reactivation.

There was no statistically significant difference in the rate of CMV reactivation between patients with lymphoma compared to patients with MM. In addition, older age (≥50 years), stage of the underlying hematological malignancy, disease status at the time of transplantation, number of lines of chemotherapy before transplantation, pre-transplant hepatitis B core IgG (HBcIgG) positivity, and CMV status prior to transplantation, did not correlate with CMV reactivation as shown in Table 3. Furthermore, prior therapy with bortezomib in patients with MM was not associated with increased risk of CMV reactivation (P = 0.09).

**Figure 1:**
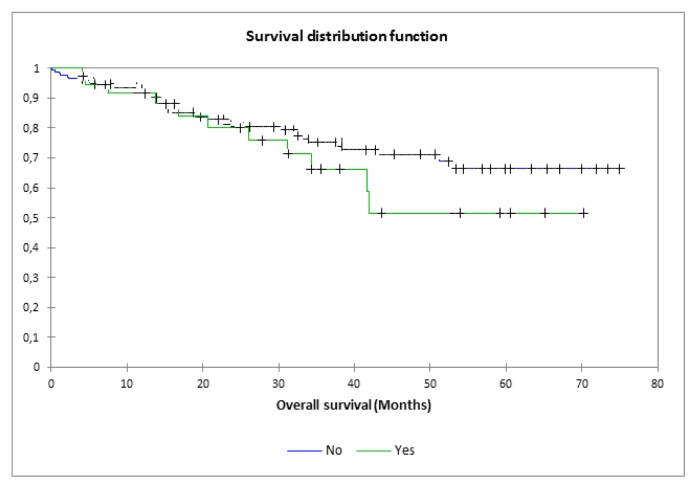
Overall survival according to CMV reactivation. Log-rank: p=0.29

Patients were followed up for a median of 32.3 (range, 3.9–75) months. The overall survival (OS) in our cohort was 76.2%, and the progression-free survival (PFS) was 55%. The log-rank test showed no difference in the OS between patients who had CMV reactivation compared to those who did not (p-values, 0.29) (Figures 1).

## Discussion

CMV reactivation is a common complication after hematopoietic stem cell transplantation, and it is more frequently reported after allogeneic versus autologous SCT. Routine monitoring of CMV reactivation following ASCT is not a universal practice among all transplant centers. The recommendations of the European Conference on Infections in Leukemia (ECIL) considered the routine surveillance for CMV reactivation after ASCT to be unnecessary because of the low likelihood of CMV disease. Nonetheless, high risk ASCT recipients, including those receiving CD34-selected grafts, and those who had prior treatment with fludarabine, cladribine, or alemtuzumab were considered to be potential candidates for CMV monitoring and the use of pre-emptive therapy.^14^

The incidence of CMV IgG positivity in our population is 90% compared to 60% in the European population.^11^ Furthermore, Han et al reported that the CMV antigenemia rate among seropositive non-transplant cancer patients was 14.3%, compared to only 2.5% among CMV seronegative patients.^8^ Our practice from 2003 to 2007 was to monitor routinely for CMV reactivation till day 100 post transplant. In 2007, we modified our practice to monitor for CMV reactivation in ASCT patients until day 42 based on our observation that patients who did not have reactivation before day 42 did not develop reactivation after that. Although none of them did receive CD34-selected graft, Alemtuzumab, Cladribine, or Fludarabine before ASCT, we didn’t stop routine monitoring of our patients considering the high incidence of CMV IgG seropositivity in our population.

In this study, despite the higher prevalence of CMV positivity, the rate of CMV reactivation (17.6%) was lower than what has been reported (30–40%) in previous studies.^2^–^10^ Differences in the study design may at least partially account for this lower-than-anticipated rate. Polymerase chain reaction (PCR) was used in some studies for CMV monitoring; this is a more sensitive test as compared to the CMV antigenemia test used in our study. Furthermore, we did not include patients with a positive CMV antigenemia test that did not meet the preset definition of CMV reactivation in our cohort.

In our cohort, the rate of CMV reactivation was higher in patients with lymphoma (20%) compared to patients with MM (14.7%). However, the univariate analysis showed no association between the underlying disease and CMV reactivation. In comparison, Rossini et al. reported higher rates of CMV reactivation in patients with MM (42%) compared to patients with lymphoma (29%).^2^ However, the effect of the underlying disease on CMV reactivation was not further analyzed in Rossini’s study.

Only 10 patients with MM of our cohort received bortezomib-based therapy before transplantation; three of which had CMV reactivation (30%) compared to 11 out of 85 (12.9%) patients who did not receive bortezomib, but the difference was not statistically significant. The lack of statistical difference in our study may in part be due to the small number of patients who received bortezomib. In a study that compared 80 patients with MM who received novel therapies prior to transplantation versus 89 patients who were treated with standard regimens, Marchesi et al reported a significantly higher rate of CMV reactivation in the former group.^15^

Kim et al.^16^ reported on the association of tandem transplantation and CMV reactivation; this was not assessed in our study due to the fact that tandem transplantation was not utilized routinely in our cohort of patients.

Marchesi et al. reported that pre-transplant HBcIgG seropositivity was a predictor of clinically relevant CMV infection in patients with lymphoma undergoing ASCT, with a 40% rate of CMV reactivation in HBcIgG-positive patients compared to 9.8% in HBcIgG-negative patients (P value, 0.008).^17^ This was not the case in our study, as the rate of CMV reactivation in the HBcIgG-positive group was 15.2%, compared to 16.9% in the negative group (P value, 0.79). This difference might be due to the low rate of CMV reactivation in our study (17.6%) compared to 40% in the Marchesi study which used PCR for monitoring rather than CMV antigenemia testing.

Previous studies have shown superiority of valacyclovir prophylaxis in comparison to acyclovir in recipients of allogeneic SCT with less CMV reactivation.^18^,^19^ This datum inspired the rationale for our strategy of switching to valacyclovir prophylaxis in patients who had a positive CMV antigenemia test that did not meet the definition of CMV reactivation in our study. In our cohort eleven patients were switched from acyclovir to valacyclovir, but despite this switch 80% of these patients had rising CMV antigenemia levels on repeated tests that required preemptive therapy.

The duration of CMV monitoring following ASCT varied in different studies. In the study by Kim et al.,^16^ CMV monitoring was done until patients started on maintenance chemotherapy after transplantation for MM, while in the study by Rossini et al., monitoring was done till day 60 post-transplantation.^2^ In our cohort, we monitored CMV reactivation until day 42 if no positive CMV antigenemia test was documented, and extended it to 2 months after the last positive test in those who had any positive CMV antigenemia test before day 42. This strategy seems to have been effective; the median time for reactivation in our study was 31 (range, 21–54) days and all cases of CMV reactivation were detected during the monitoring period. This finding is similar to that reported by Marchesi et al.^15^ where the median time to CMV reactivation was 33 days.

In our cohort, treatment of CMV reactivation followed the usual standards with ganciclovir and valganciclovir being used in the majority of patients (94.6%) as the initial preemptive therapy. Furthermore, all patients who had CMV reactivation cleared their CMV antigenemia with the first line of therapy, and no patient developed symptomatic CMV infection.

It is interesting to note that CMV reactivation did not affect the overall survival of patients in our cohort. Moreover, none of the patients who developed CMV reactivation died in the first 100 days post transplantation.

This study has some limitations inherent to its design. It is a retrospective study and from a single cancer center. Therefore, our findings may not apply to other centers with different patient populations. Also, testing for CMV reactivation was carried out by the CMV antigenemia test only with no utilization of PCR. Finally, most of the patients in our cohort received standard chemotherapeutic regimens prior to transplantation, so the effect of different modalities of treatments used before transplantation, especially novel therapies, on the CMV reactivation could not be carefully assessed.

In conclusion, in our cohort of lymphoma and MM patients, the rate of CMV reactivation following ASCT was 17.6%. There was no difference in reactivation rates between lymphoma and MM patients. With the use of preemptive therapy, symptomatic CMV infection was not documented in any patient in our cohort. Monitoring of CMV antigenemia until day 42 post-transplantation seemed to be effective in patients who did not have any positive CMV antigenemia test. CMV reactivation had no impact on patients’ survival post ASCT.

## Figures and Tables

**Table 1:** Clinical characteristics of 210 patients who underwent autologous peripheral blood stem cell transplantation.

Patient characteristics	Number (%)
Total	210 (100%)
Age, years – Median (range)	43 (19–65)
Age Groups	
18–29	51 (24.3%)
30–39	34 (16.2%)
40–49	45 (21.4%)
50–59	57 (27.1%)
≥60	23 (11.0%)
Gender	
Male	87 (41.4 %)
female	123 (58.6 %)
Underlying disease	
Lymphoma	115 (54.8 %)
Multiple myeloma	95 (45.2 %)
Lines of chemotherapy before transplantation	
≤3 lines	182(86.6%)
>3 lines	25 (12%)
unknown	3(1.4%)
Disease status before transplant	
Complete remission	34 (16.1 %)
Partial remission	147 (70.0 %)
Stable disease	4 (2.0 %)
unknown	25 (11.9 %)
CMV status before transplant	
IgG positive	205 (97.6%)
IgM positive	10 (4.8 %)
CMV prophylaxis given	
Acyclovir	198 (94.3%)
Valacyclovir	11 (5.2%)
Unknown	1 (0.5%)
Conditioning regimen	
Lymphoma patients	
BEAM	111 (96.5%)
TEAM	4 (3.5%)
Multiple myeloma patients	
Melphalan	95 (100%)
Median time to engraftment, days (range)	
Median neutrophil engraftment*	10 (7–15)
Median platelet engraftment**	16 (7–47)
Mortality	
Overall	50 (23.8%)
Day 100	6 (2.9%)

Abbreviations: CMV, Cytomegalovirus; BEAM, BCNU, etoposide, cytarabine, melphalan; TEAM, thiotepa, etoposide, cytarabine, melphalan.

* Absolute neutrophil count > 500 ×10^9^/L for 2 consecutive days.

** Platelet count > 20 ×10^9^/L.

**Table 2:** Clinical characteristics, management and outcomes of patients with CMV reactivation.

Modality	Number (%)
CMV reactivation	
Yes	37 (17.6%)
No	173 (82.4%)
Time to reactivation, days	
Median (range)	31 (21–54)
CMV antigenemia levels*	
Median (range)	6 (3–26)
Anti-CMV therapy	
Ganciclovir	26 (70.3%)
Valganciclovir	9 (24.3%)
Foscarnet	2 (5.4%)
Treatment-related complications	
Pancytopenia	3 (8.1%)
Neutropenia	5 (13.5%)
Thrombocytopenia	4 (10.8%)
Renal impairment	2 (5.4%)
Hypomagnesaemia	5 (13.5%)
Hypocalcemia	1 (2.7%)
Other complications	3 (8.1%)
Duration of anti-CMV therapy, days	
Induction therapy, median (range)	8 (4–15)
Maintenance therapy, median (range)	10 (6–16)
Development of CMV disease	
Yes	0 (0%)
No	37 (100%)
Mortality	
Overall	11 (29.7%)
Day 100	0

* Positive cell/250,000 leukocyte.

**Table 3:** Comparison of the clinical characteristics of patients who developed CMV reactivation versus those who did not.

Modality	Total number 210	CMV reactivation		P-value

		Yes Number (%)	No Number (%)	
Age groups				
<50 years	130 (61.9%)	25 (19.2%)	105 (81.5%)	0.44
≥50 years	80 (38.1%)	12 (15.0%)	68 (85.0%)	
Underlying disease				
Lymphoma	115 (55%)	23(20.0%)	92(80.0%)	0.32
Multiple myeloma	95 (45%)	14(14.7%)	81(85.3%)	
Stage of the underlying disease				
Stage I/II	100(48%)	14(14.0%)	86(86.0%)	0.23
Stage III/IV	82(39%)	17(20.7%)	65(79.3%)	
Unknown	28(13%)	6(21.4%)	22(78.6%)	
Lines of chemotherapy before transplantation				
≤3 lines	182(86.6%)	33(18.1%)	149(81.9%)	0.45
>3 lines	25(12%)	3(12.0%)	22(88.0%)	
Unknown	3(1.4%)	1(33.3%)	2(66.7%)	
Receipt of bortezomib before transplantation (multiple myeloma only)				
Yes	10 (10.5%)	3 (30%)	7 (70%)	0.09
No	85 (89.5%)	11 (12.9%)	74 (87.1%)	
Disease status before transplant				
Complete remission	34 (16%)	7 (20.6%)	27 (79.4%)	0.59
Partial response	147 (70%)	26 (17.7%)	121 (82.3%)	
Stable disease	4 (2%)	0	4 (100%)	
Unknown	25 (12%)	4 (16 %)	21 (84%)	
CMV status before transplant				
IgG positive	205 (97.6%)	37(18.0%)	168(82.0%)	0.30
IgM positive	10 (5%)	3(30.0%)	7(70.0%)	0.29
HBcIgG seropositivity				
Positive	33 (15.7%)	5 (15.2%)	28 (84.2%)	0.79
Negative	177 (84.3%)	30 (16.9%)	147 (83.1%)	

Abbreviations: CMV, Cytomegalovirus; HBcIgG, hepatitis B core IgG.
